# Combined Impact of Lifestyle Factors on Prospective Change in Body Weight and Waist Circumference in Participants of the EPIC-PANACEA Study

**DOI:** 10.1371/journal.pone.0050712

**Published:** 2012-11-30

**Authors:** Anne M. May, Dora Romaguera, Noémie Travier, Ulf Ekelund, Manuela M. Bergmann, Rudolf Kaaks, Birgit Teucher, Annika Steffen, Heiner Boeing, Jytte Halkjaer, Anne Tjonneland, Marianne Uhre Jakobsen, Kim Overvad, Laureen Dartois, Guy Fagherazzi, Marie-Christine Boutron-Ruault, J. Ramón Quirós, Antonio Agudo, Carlos Gonzalez, María-José Sánchez, Pilar Amiano, Jose-Maria Huerta, Eva Ardanaz, Nicholas J. Wareham, Francesca L. Crowe, Androniki Naska, Philippos Orfanos, Antonia Trichopoulou, Domenico Palli, Claudia Agnoli, Rosario Tumino, Paolo Vineis, Salvatore Panico, H. Bas Bueno-de-Mesquita, Monique Verschuren, Isabel Drake, Emily Sonestedt, Tonje Braaten, Sabina Rinaldi, Isabelle Romieu, Nadia Slimani, Teresa Norat, Elio Riboli, Petra H. M. Peeters

**Affiliations:** 1 Julius Center for Health Sciences and Primary Care, University Medical Center Utrecht, Utrecht, The Netherlands; 2 National Institute for Public Health and the Environment (RIVM), Bilthoven, The Netherlands; 3 Department of Epidemiology & Biostatistics, School of Public Health, Imperial College London, London, United Kingdom; 4 Unit of Nutrition, Environment and Cancer, Catalan Institute of Oncology, Barcelona, Spain; 5 Medical Research Council, Epidemiology Unit, Institute of Metabolic Science, Cambridge, United Kingdom; 6 Department of Epidemiology, German Institute of Human Nutrition, Potsdam-Rehbruecke, Germany; 7 Division of Clinical Epidemiology, German Cancer Research Center, Heidelberg, Germany; 8 Institute of Cancer Epidemiology, Danish Cancer Society, Copenhagen, Denmark; 9 Department of Clinical Epidemiology, Aarhus University Hospital, Aarhus, Denmark; 10 Department of Cardiology, Center for Cardiovascular Research, Aalborg Hospital, Aarhus University Hospital, Aalborg, Denmark; 11 INSERM, CESP Centre for Research in Epidemiology and Population Health, Institut Gustave Roussy, Villejuif, France; 12 Paris-Sud University, Villejuif, France; 13 Public Health and Participation Directorate, Asturias, Spain; 14 Andalusian School of Public Health, Granada, Spain; 15 CIBER Epidemiology and Public Health CIBERESP, Barcelona, Spain; 16 Public Health Division of Gipuzkoa, Basque Government, Spain; 17 Department of Epidemiology, Regional Health Authority, Murcia, Spain; 18 Navarra Public Health Institute, Pamplona, Spain; 19 Cancer Epidemiology Unit, University of Oxford, Oxford, United Kingdom; 20 WHO Collaborating Center for Food and Nutrition Policies, Department of Hygiene, Epidemiology and Medical Statistics, University of Athens Medical School, Athens, Greece; 21 Hellenic Health Foundation, Athens, Greece; 22 Molecular and Nutritional Epidemiology Unit, CSPO-Scientific Institute of Tuscany, Florence, Italy; 23 Nutritional Epidemiology Unit, IRCCS Foundation, National Cancer Institute, Milan, Italy; 24 Cancer Registry and Histopathology Unit, “Civile - M.PArezzo” Hospital, Ragusa, Italy; 25 HuGeF Foundation, Torino, Italy; 26 Dipartimento di Medicina Clinica e Sperimentale Federico il University, Naples, Italy; 27 Department of Gastroenterology and Hepatology, University Medical Center Utrecht, Utrecht, The Netherlands; 28 Department of Clinical Sciences in Malmö, Lund University, Lund, Sweden; 29 Institute of Community Medicine, University of Tromsø, Tromsø, Norway; 30 International Agency for Research on Cancer (IARC-WHO), Lyon, France; Brigham and Women’s Hospital and Harvard Medical School, United States of America

## Abstract

**Background:**

The evidence that individual dietary and lifestyle factors influence a person’s weight and waist circumference is well established; however their combined impact is less well documented. Therefore, we investigated the combined effect of physical activity, nutrition and smoking status on prospective gain in body weight and waist circumference.

**Methods:**

We used data of the prospective EPIC-PANACEA study. Between 1992 and 2000, 325,537 participants (94,445 men and 231,092 women, aged between 25–70) were recruited from nine European countries. Participants were categorised into two groups (positive or negative health behaviours) for each of the following being physically active, adherent to a healthy (Mediterranean not including alcohol) diet, and never-smoking for a total score ranging from zero to three. Anthropometric measures were taken at baseline and were mainly self-reported after a medium follow-up time of 5 years.

**Results:**

Mixed-effects linear regression models adjusted for age, educational level, alcohol consumption, baseline body mass index and follow-up time showed that men and women who reported to be physically active, never-smoking and adherent to the Mediterranean diet gained over a 5-year period 537 (95% CI −706, −368) and 200 (−478, −87) gram less weight and 0.95 (−1.27, −0.639) and 0.99 (−1.29, −0.69) cm less waist circumference, respectively, compared to participants with zero healthy behaviours.

**Conclusion:**

The combination of positive health behaviours was associated with significantly lower weight and waist circumference gain.

## Introduction

General and abdominal adiposity are related to mortality and many adverse health-related outcomes, such as diabetes, cardiovascular diseases, and several types of cancer [1]–[4]. Maintaining a healthy body weight is therefore desirable. The evidence that individual modifiable lifestyle behaviours, such as physical activity, smoking and diet influence a person’s weight is well established [5]–[8]. In previous studies, we have investigated the association of each individual lifestyle behaviour separately and subsequent changes of weight and waist circumference in a large cohort of European adults participating in the EPIC-PANACEA (European Prospective Investigation into Cancer-Physical Activity, Nutrition, Alcohol, Cessation of Smoking, Eating out of home And obesity) project. Our results showed that physical activity was inversely associated with both general and abdominal adiposity in cross-sectional analyses, whereas higher levels of baseline physical activity predicted lower gain in abdominal but not general adiposity [9], [10]. Furthermore, men and women who adhere to a Mediterranean diet, high in foods of vegetable origin and unsaturated fatty acids, had a lower waist circumference and a 10% lower risk of developing overweight in the future compared to individuals with lower adherence [11], [12]. Moreover, although current smokers tended to weigh less they did not necessarily have a lower waist circumference than never smokers. Smoking cessation tended to be associated with weight gain, however weight gain in individuals who stopped smoking at least 1 year before recruitment was comparable to never smokers’ gain [13], [14].

Given these associations observed with individual health behaviours, we were interested in investigating the combined impact of these behaviours on subsequent changes in general and abdominal obesity. Studying the combined effect will provide insight on the potential benefits of adopting not just one but a range of healthy lifestyle habits. It has been shown that the combined impact of health behaviours was associated with a reduced risk of major chronic diseases including myocardial infarction, stroke, diabetes mellitus and cancer and was also inversely related to mortality [15]–[26]. The combined effect of health behaviours on obesity, which is an intermediate risk factor of many chronic diseases and mortality, has rarely been studied. In two cross-sectional studies positive health behaviours were associated to less subcutaneous and visceral adipose tissue [27] and lower BMI and waist circumference [28] compared to participants with less health behaviours. Moreover, Mozaffarian et al. [29] showed that weight gain was lowest in the participants with positive changes of diet and physical activity.

The aim of the present study was to investigate the combined effect of physical activity, smoking status, and diet on future weight and waist circumference gain by combining these behaviours into a simple health behaviour score in the large EPIC-PANACEA study.

## Methods

From 1992–2000, in the EPIC study more than 500,000 individuals aged between 25 and 70 years were recruited from 23 centres in 10 countries (Denmark, France, Germany, Greece, Italy, the Netherlands, Norway, Spain, Sweden, and the United Kingdom) [30], [31]. Approval for this study was obtained from the ethical review boards of the International Agency for Research on Cancer and from local institutions.

Of the total cohort of 519,931 participants, we excluded 22,196 individuals because of missing dietary and non-dietary questionnaires, extreme energy intake to energy expenditure ratio, pregnant women, and missing baseline weight or extreme anthropometry. Furthermore, we excluded participants with missing follow-up weight (n = 121,853; this included the cohorts of Turin and Ragusa (both Italy) and parts of cohorts from Norway and Naples (Italy)) or extreme anthropometry at follow-up (i.e., weight change<−5 or >5 kg/year over several years (n = 1,926) or BMI<16 kg/m^2^ at follow-up (n = 140)). Information on smoking, physical activity or dietary variables was not available for 48,279 participants. Physical activity was mainly missing because centres in Norway and Umea (Sweden) used different questionnaires to assess physical activity. Therefore, the final study population comprised 325,537 participants from nine countries.

A total of 88,972 participants from Florence (Italy), Potsdam (Germany), Doetinchem (Netherlands) and Cambridge (United Kingdom) also provided data on waist circumference at baseline and follow-up.

### Anthropometric Measures

Baseline body weight (kg) and height (cm) were measured according to standardised procedures without shoes [32], except for the centres of Oxford (UK) and France where self-reported anthropometric values at baseline were used. Baseline waist circumference (cm) was measured either at the narrowest torso circumference or at the midpoint between the lower ribs and iliac crest. Weight and waist measurements were corrected to account for protocol differences between centres as previously described [32]. Briefly, for subjects who were dressed normally and were without shoes, 1.5 kg for weight and 2.0 cm for circumferences were subtracted from the original measurement, whereas for subjects in light clothing without shoes, 1 kg was subtracted from the weight.

At follow-up, weight and waist circumference were self-reported in all centres except in Cambridge (UK) and Doetinchem (Netherlands) where it was measured according to the baseline protocol. BMI was calculated as body weight (kg) divided by height squared (m^2^). For descriptive purposes, the accuracy of self-reported anthropometric measures was improved with the use of prediction equations derived from subjects with both measured and self-reported values (i.e., the Oxford correction equations) [33].

As follow-up times between first and second anthropometry assessment differed by centre (2–11 years), our main outcome was the 5-year change (i.e. ((follow-up weight or waist circumference -baseline weight or waist circumference)/years of follow-up)*5).

### Health Behaviour Score

We investigated the combined effect of three lifestyle factors, that is physical activity, diet (according to a Mediterranean diet that is associated to weight change [12]) and smoking using a pragmatic health behaviour score. All lifestyle factors were assessed at baseline. Smoking status was also assessed at follow-up in a subpopulation of 288,167 participants.

Habitual physical activity was self-reported using a standardized questionnaire and a validated 4-category index was derived by cross-classifying three questions referring to activities during the last year against classification of work activity [34]. For the current purpose, we dichotomised the index into inactive (sedentary job and no recreational activity) and active (any category with activity levels above the latter).

Usual food intakes were measured using country-specific validated dietary questionnaires [30]. The modified Mediterranean Diet Score (mMDS) [35], [36], which is a variant of the original MDS [37] and has an applicability in both Mediterranean and non-Mediterranean countries, was constructed as described elsewhere [11], [36], [37] with the exception that alcohol consumption was not included in the score. The mMDS scored the consumption of 8 components of the Mediterranean diet (high fruit, vegetable, legumes, fish, cereals, unsaturated to saturated fat ratio, and low meat & meat products and dairy products). The score could take a value from 0 (minimal adherence) to 8 (maximal adherence), and was further categorized as low (0–4 points) and high (5–8 points) adherence to the mMDS, based on the median consumption of each of the indicated components observed in the present population.

Information on smoking status (never, former, current) at recruitment was assessed with standard questionnaires. For the current purpose, participants were grouped in two categories: ever smokers (current and former) and never smokers. In a subpopulation of 288,167 participants also information on smoking status at follow-up was available [14].

We constructed a simple pragmatic health behaviour score. Participants could get one point for each health behaviour, i.e., physically active, high adherence to the mMDS and never-smoking for a total score ranging from 0–3. Comparable scores were previously used and were associated with reduced risk of major chronic diseases [15], [17]–[26].

### Covariates

Total amount of daily consumed alcohol over the last 12 months was assessed by country-specific validated dietary questionnaires [30] and summarized as non-consumers, 1–6 g/day, 7–18 g/day, 19–30 g/day, 31–60 g/day, and >60 g/day (women) and 61–96 g/day and >96 g/day (men). Total energy intake was computed from the dietary questionnaire. The educational level (none, primary school, technical school, secondary school, and university degree) was used as a proxy for socioeconomic status. Information on the presence of chronic diseases (heart disease, stroke, diabetes mellitus, and cancer) before or at recruitment was assessed by questionnaire.

### Statistical Analyses

Baseline characteristics of the study population are presented by health behaviour score and gender by using mean and standard deviation for continuous variables and percentages for categorical variables.

Associations between the health behaviour score and 5-year weight and waist circumference changes were modelled using sex-specific multilevel random mixed-effects linear regression, taking clustering of participants within countries and centres into account. Analyses were adjusted for age (years, continuous), follow-up time (years, continuous), baseline BMI (kg/m^2^, continuous), education (categorical), total energy intake (kcal, continuous), and alcohol intake (categorical). The model with 5-year waist circumference change as the outcome was additionally adjusted for baseline waist circumference (cm, continuous) and in a second model also for BMI change (continuous) for estimation of the relationship between the health behaviour score and waist circumference change independently of changes of general obesity.

Plausible effect modification by age (<60 and ≥60 years of age), baseline BMI (<25, 25–30 and ≥30 kg/m^2^), and education were explored by adding a product term. Stratified analyses were conducted when the product term was significant.

Random-effect meta-analysis was used to assess whether there was heterogeneity among countries. Country specific estimates were calculated by using general linear models in countries with one centre only and multilevel mixed-effects models in countries with more than one centre.

Sensitivity analyses were performed by excluding participants with chronic diseases at baseline as well as participants who quit smoking during follow-up. Furthermore, we included never smoking as the health behaviour in the score. Since never smoking is a non-modifiable factor and current smokers can only revert to former smokers, we repeated our main analyses including smoking as non-current (1 point) and current (0 point) in the health score.

Results were computed using SAS 9.2. STATA 11 was used for the meta-analyses.

## Results

In the current population, mean (standard deviation) 5-year weight gain was 0.71 (5.14) kg; when we applied the Oxford correction equations to improve the accuracy of the self-reported measurements, 5-year weight gain was 2.08 (5.07) kg. Five-year increase in waist circumference was 3.80 (6.42) cm (uncorrected) and 6.17 (6.48) cm (corrected). A total of 11.2% of men and 19.3% of women were scored to have all three health behaviours at study recruitment (Table 1). These men and women were on average younger, higher educated and had a lower alcohol intake, while their crude energy intake was higher in comparison to the participants who reported no health behaviours.

**Table 1:** Characteristics of the population stratified according to sex and the health behaviour score (n = 325,537)^1^.

Men (n = 94,445)					Women (n = 231,092)			
Heath behaviour score N (%)	0	1	2	3	0	1	2	3
	6,595 (7.0)	39,533 (41.9)	37,698 (39.9)	10,619 (11.2)	10,310 (4.5)	17,149 (30.8)	104,919 (45.4)	44,714 (19.3)
Age (years)	57.6 (8.6)	54.7 (8.6)	52.4 (9.4)	50.8 (10.1)	53.9 (9.9)	52.6 (9.2)	52.2 (9.4)	50.7 (9.6)
Baseline BMI(kg/m2)	26.8 (3.8)	26.7 (3.5)	26.8 (3.5)	26.3 (3.6)	25.5 (4.7)	25.0 (4.3)	25.1 (4.5)	24.5 (4.3)
Baseline WC (cm)	97.0 (10.4)	95.4 (10.0)	94.8 (9.9)	93.2 (9.7)	81.6 (11.8)	80.6 (11.0)	81.1 (11.6)	79.7 (11.7)
Uncorrected weight gain (kg/5 y)^2^	0.28 (5.5)	0.15 (5.4)	0.15 (5.0)	0.25 (4.7)	0.86 (5.6)	0.84 (5.3)	0.89 (5.0)	1.2 (4.8)
Corrected weight gain (kg/5 y)^3^	2.1 (5.5)	2.2 (5.3)	2.2 (5.0)	2.1 (4.6)	2.0 (5.6)	2.1 (5.3)	2.0 (5.0)	2.1 (4.7)
Uncorrected WCgain (cm/5 y)^2, 4^	2.3 (6.2)	2.5 (5.7)	2.5 (5.4)	2.6 (5.5)	4.6 (7.1)	5.0 (7.0)	4.7 (6.6)	4.0 (6.5)
Corrected WC gain (cm/5 y)^3^	6.3 (7.0)	7.5 (6.3)	7.1 (6.1)	6.6 (6.4)	5.0 (6.8)	5.7 (6.6)	5.4 (6.3)	4.8 (6.3)
Physically active (%)^5^	0	78.6	93.2	100	0	66.6	80.1	100
Never-smoking (%)	0	6.4	44.9	100	0	23.4	76.3	100
High mMDS (%)^6^	0	15.0	62.0	100	0	10.0	42.8	100
Alcoholconsumption (g/day)	24.2 (27.7)	25.3 (26.3)	23.2 (25.2)	19.0 (22.3)	10.3 (15.0)	10.6 (13.8)	8.4 (11.6)	7.7 (10.6)
Energy intake (kcal)	2297.9 (615.0)	2406.8 (639.1)	2488.2 (663.8)	2499.2 (659.3)	1885.7 (524.6)	1930.7 (519.3)	1996.6 (536.2)	2105.3 (553.7)
Highly educated(%)^7^	27.5	26.8	28.1	31.9	15.6	22.3	24.1	28.3

*Abbreviation: WC – waist circumference, mMDS – modified Mediterranean diet score.*

1 Means (standard deviation) are presented for continuous variables and percentages are presented for categorical variables.

2 Uncorrected 5-y change means that self-reported weight or waist circumference at either baseline or follow-up (for individuals with no measured weight available) was used to calculate weight change.

3 Corrected 5-y change means that the “Oxford correction equations” [33] were applied to individuals with self-reported weight at either baseline or follow-up to predict their likely measured weight, and to calculate weight change.

4 Waist circumference is available for a subsample of 37,245 men and 51,727 women.

5 Physically active is defined as a Cambridge Physical Activity Index [51] of >1, i.e., not being inactive that is if a person has a sedentary occupation and perform at least half an hour of leisure time activity a day, such as cycling or swimming; or else a non-sedentary occupation with or without leisure time activity.

6 High mMDS includes person with a mMDS score above the median.

7 Highly educated is defined as a longer education than secondary school (including an university degree).

Table 2 shows the independent effects of the individual health behaviours. Participants who were physically active, never-smoking, or highly adherent to the mMDS had on average a lower 5-year weight gain compared to people who were inactive, smoking or low adherent to the mMDS. However, relations were only significant for never-smoking and low mMDS adherent men or for physically active women. Five-year gain in waist circumference was lower for people who were physically active, never-smoking, and for women reporting high mMDS scores.

**Table 2:** Independent association between individual health behaviours and weight change (kg/5 y) and waist circumference change (cm/5 y) in EPIC-PANACEA participants.

	Weight change (kg/5 y)^1^		Waist circumference change (cm/5 y) 2	
	ß	95% CI	ß	95% CI
**Men**				
Physical active vs inactive	−0,073	(−0.158; 0.012)	−0,465	(−619; −0.311)
Never smoker vs ever smoker	−0,335	(−0.405; −0.266)	−0,469	(−0.589; −0.350)
High adherence to mMDS vs low adherence	−0,090	(−0.175; −0.005)	−0,047	(−0.093; 0.186)
**Women**				
Physical active vs inactive	−0,078	(−0.129; −0.026)	−0,276	(−0.419; −0.133)
Never smoker vs ever smoker	−0,018	(−0.061; 0.028)	−0,413	(−0.520; −0.306)
High adherence to mMDS vs low adherence	−0,031	(−0.078; 0.016)	−0,234	(−0.375; −0.102)

*Abbreviations: CI – confidence interval, mMDS – modified Mediterranean Diet Score,*

1 Linear mixed models adjusted for age, total energy intake, baseline body mass index, education, alcohol intake and follow-up time (men: n = 94,445; women n = 231,092). Individual health behaviours are also adjusted for each other.

2 Linear mixed models adjusted for age, total energy intake, baseline body mass index, baseline waist circumference, education, alcohol intake and follow-up time (men: n = 37,245; women n = 51,727). Individual health behaviours are also adjusted for each other.

Table 3 shows the adjusted relationships between the health behaviour score and 5-year weight and waist circumference change. For each unit increase in the score, men and women gained significantly less weight and waist circumference (all p-values for trend <0.0001). Compared to those with zero health behaviour, men who reported all three health behaviours gained 537 grams (confidence interval −706; −368) less weight and 0.95 cm (−1.27, −0.63) less waist circumference. Women with three health behaviours gained 200 grams (−278; −87) and 0.986 cm (−1.29; −0.69) less weight and waist circumference, respectively. Additional adjustment for simultaneous change of BMI in the model with waist circumference change as the outcome only slightly attenuated the results (−0.69 cm (−0.95; −0.42) and −0.68 cm (−0.93; −0.42) for men and women with three health behaviours versus zero, respectively (both p-values for trend<0.0001)).

**Table 3:** Association between the health behaviour score and weight change (kg/5 y) and waist circumference change (cm/5 y) in EPIC-PANACEA participants.

	Weight change (kg/5 y)^1^		Waist circumference (cm/5 y)^2^	
	ß (95% confidence interval)		P for trend	ß (95% confidence interval)		P for trend
**Health behaviour score**						
** Men**						
** 0**	0		<0.0001	0		<0.0001
** 1**	−0.136	(−0.274; 0.002)		−0.491	(−0.708; −0.273)	
** 2**	−0.338	(−0.480; −0.197)		−0.758	(−0.986; −0.530)	
** 3**	−0.537	(−0.706; −0.368)		−0.948	(−1.267; −0.630)	
** Women**						
** 0**	0		<0.0001	0		<0.0001
** 1**	−0.171	(−0.278; −0.064)		−0.386	(−0.636; −0.136)	
** 2**	−0.210	(−0.316; −0.105)		−0.719	(−0.968; −0.471)	
** 3**	−0.200	(−0.278; −0.087)		−0.986	(−1.286; −0.687)	

1 Linear mixed models adjusted for age, total energy intake, baseline body mass index, education, alcohol intake and follow-up time (men: n = 94,445; women n = 231,092).

2 Linear mixed models adjusted for age, total energy intake, baseline body mass index, baseline waist circumference, education, alcohol intake and follow-up time (men: n = 37,245; women n = 51,727).

For changes of weight and waist circumference, significant interactions were found between the health behaviour score and baseline BMI, except in women when waist circumference change was the outcome. Stratified analyses showed that the relationship between the score and weight change was stronger for people with normal weight (BMI<25) and less obvious for obese people (BMI≥30) (Table 4). The relationship with waist circumference change was more consistent for all BMI categories, with largest gain in obese women. No significant interactions between age or education and the health behaviour score were observed, except for younger and older women and weight change. However, results were similar for women aged below or above 60 years (data not shown).

**Table 4:** Subgroup analysis stratified baseline body mass index for the association between the health behaviour score and weight change (kg/5 y) and waist circumference change in EPIC-PANACEA participants.

Heath behaviorscore	N	0	1		2		3		P for trend
		ß	ß	95% CI	ß	95% CI	ß	95% CI	
**Weight change (kg/5** **y)** 1									
**Men**									
BMI<25 kg/m2	31,425	0	−0.176	(−0.380; 0.029)	−0.463	(−0.672; −0.255)	−0.669	(−0.914; −0.425)	<0.0001
BMI 25–30 kg/m2	47,558	0	0.034	(−0.157; 0.225)	−0.167	(−0.363; 0.029)	−0.368	(−0.604; −0.133)	<0.0001
BMI≥30 kg/m2	15,396	0	−0.462	(−0.892; −0.031)	−0.504	(−0.947; −0.061)	−0.535	(−1.079; 0.31)	0.095
**Women**									
BMI<25 kg/m2	135,739	0	−0.197	(−0.321; −0.072)	−0.232	(−0.355; −0.110)	−0.210	(−0.340; −0.079)	0.028
BMI 25–30 kg/m2	65,849	0	−0.030	(−0.238; 0.177)	0.014	(−0.190; 0.219)	0.026	(−0.197; 0.249)	0.41
BMI≥30 kg/m2	29,382	0	−0.350	(−0.724; 0.024)	−0.376	(−0.744; −0.007)	−0.350	(−0.757; 0.056)	0.25
**WC change (cm/5** **y)** 2									
**Men**									
BMI<25 kg/m2	13,340	0	−0.507	(−0.859; −0.156)	−0.904	(−1.269; −0.539)	−1.078	(−1.551; −0.605)	<0.0001
BMI 25–30 kg/m2	18,984	0	−0.434	(−0.753; −0.154)	−0.660	(−0.976; −0.345)	−0.913	(−1.362; −0.465)	<0.0001
BMI≥30 kg/m2	4,888	0	−0.640	(−1.290; 0.009)	−0.757	(−1.453; −0.031)	−0.310	(−1.509; 0.089)	0.13
**Women**									
BMI<25 kg/m2	27,346	0	−0.336	(−0.659; −0.013)	−0.618	(−0.940; −0.300)	−0.902	(−1.280; −0.524)	<0.0001
BMI 25–30 kg/m2	17,529	0	−0.280	(−0.733; 0.173)	−0.615	(−1.065; −0.166)	−0.735	(−1.281; −0.189)	<0.0001
BMI≥30 kg/m2	6,806	0	−0.960	(−1.686; −0.233)	−1.573	(−2.294; −0.851)	−2.091	(−3.023; −1.159)	<0.0001

*Abbreviations: CI – confidence interval, BMI – body mass index, WC – waist circumference*.

1 Linear mixed models adjusted for age, total energy intake, baseline body mass index, education, alcohol intake and follow-up time.

2 Linear mixed models adjusted for age, total energy intake, baseline body mass index, baseline waist circumference, education, alcohol intake and follow-up time.

Heterogeneity in the association between the health behaviour score and anthropometry changes between countries was assessed using the meta-analysis approach (Appendix Figures S1, S2, S3, S4). For men, no heterogeneity was present (p for heterogeneity>0.05). For women a significant degree of heterogeneity was found for the association with body weight and (I^2^ = 60.7% (p = 0.002)) waist circumference change (I^2^ = 62.0% (p = 0.033)). In most countries, the associations between the health behaviour score and anthropometry changes were inverse, with the exception for Cambridge and Oxford-Health Conscious (United Kingdom), and Greece where non-significant positive associations with weight change were observed.

Excluding participants with chronic diseases at baseline did not change the results (data not shown).

In 288,167 participants information on smoking habits at follow-up was available. Twenty-two percent of baseline smokers quit smoking during follow-up. Excluding these quitters from the analyses resulted in an attenuation of the effect of the health behaviour score on weight in men (three health behaviours versus zero: −298 grams (−0.483; −0.112) and a null effect in women (2 grams (−0.123; 0.127) (Table 5). Results for waist circumference change were also attenuated in men (0.70 cm (−1.02; −0.37)) and women (−0.75 cm (−1.06; −0.44)).

**Table 5:** Sensitivity analyses: Association between the health behaviour score and weight change (kg/5 y) and waist circumference change (cm/5 y) excluding EPIC-PANACEA participants who quit smoking during follow-up^1.^

	Weight change (kg/5 y)^2^		Waist circumference (cm/5 y)^3^	
	ß (95% confidence interval)		P for trend	ß (95% confidence interval)		P for trend
**Health behaviour score**						
** Men**						
** 0**	0		0.0004	0		<0.0001
** 1**	−0.103	(−0.253; 0.048)		−0.410	(−0.639; −0.182)	
** 2**	−0.159	(−0.313; −0.005)		−0.541	(−0.779; −0.304)	
** 3**	−0.298	(−0.483; −0.112)		−0.697	(−1.021; −0.372)	
** Women**						
** 0**	0		0.06	0		<0.0001
** 1**	−0.070	(−0.188; 0.049)		−0.318	(−0.584; −0.052)	
** 2**	0.002	(−0.115; 0.118)		−0.505	(−0.768; −0.242)	
** 3**	0.002	(−0.123; 0.127)		−0.747	(−1.058; −0.436)	

1 Information about smoking status at follow-up was available for 288,167 participants (81,227 men and 206,940 women).

2 Linear mixed models adjusted for age, total energy intake, baseline body mass index, education, alcohol intake and follow-up time (men: n = 81,227; women n = 206,940).

3 Linear mixed models adjusted for age, total energy intake, baseline body mass index, baseline waist circumference, education, alcohol intake and follow-up time (men: n = 36,949; women n = 51,221).

Sensitivity analyses including smoking as non-current (1 point) and current (0 point) in the health score yielded results comparable to our main analyses: Men and women who reported to be physically active, non-smoking and adherent to the Mediterranean Diet gained lesser weight and waist (men: −469 grams (95% CI: −674, −0.264) and women: −331 grams (−480, −182) and men: −0.93 cm (−1.27, −0.59) and women: −1.18 cm (−1.55, −0.83), respectively) compared to participants with no healthy behaviours.

## Discussion

In the present large prospective study, the combination of positive health behaviours was associated with significantly lower weight gain and smaller increase in waist circumference in a dose-response manner. Men and women who reported to be physically active, never-smoking and adherent to the Mediterranean Diet (mMDS) gained over a 5-year period on average 537 and 200 grams less weight and 0.95 and 0.99 cm less waist circumference, respectively, as compared to men and women who reported to be physically inactive, ever smokers and not adherent to the mMDS.

Our results confirm findings from studies investigating individual relationships between physical activity, smoking and diet and changes in general and abdominal adiposity showing that each individual behaviour is related with changes in adiposity [10], [12], [13], [27], [29], [38]–[40]. We extend these findings by reporting that combining these health behaviours leads to a lower increase in general and central adiposity than each single health behaviour. Bullo et al. [28] and Molenaar et al. [27] also investigated the effect of combined health behaviours and showed that reporting more health behaviours was cross-sectionally related to lower general and abdominal adiposity. However, the relationship with prospective changes was not investigated and the latter did not take diet into account.

In contrast to others [18]–[28] who investigated the association between combined health behaviours and body weight, we did not include moderate alcohol consumption as a health behaviour in our score. Although moderate alcohol consumption was shown to be associated to lower vascular risk [41], any use of alcohol is related to increased cancer risk [42]. Alcohol abuse causes many health-related harms [43]. Thus, in terms of public health, we prefer not to include alcohol as a potential beneficial behaviour and instead adjust for alcohol consumption in the analyses.

Combining the health behaviours in one score led to stronger associations compared to the associations of the individual behaviours with weight and waist circumference changes we reported earlier [10], [12]. This is in line with Mouzaffarian el al. [29] who showed modest individual associations of diet and physical activity with weight changes and stronger associations in the aggregated analyses.

Recently in controlled intervention studies, it was reported that physical activity did not further affect weight loss in addition to a diet [44], [45]. However, these trials measure only short-term effects, generally include a selected population (e.g. motivated to lose weight and to follow an intervention, only obese participants or health conscious participants). It might also be that the diet+exercise group compensates the extra activity with more sedentary time or a higher caloric intake. Results of a prospective cohort-study among free-living populations with long follow-up might provide results that rather reflect the real world, although residual confounding cannot be ruled out.

Strengths of the present study are the prospective design, the large sample of participants from 9 European countries and the use of validated and standardized questionnaires.

A limitation of our study was that in most of the centres weight and waist circumference at second assessment were self-reported and were therefore possibly underreported [46]. Generally, self-reported weight and waist circumference appear reasonably valid [47]. High correlations were reported between self-reported and measured weight and waist circumference in the present population as well (all Spearman correlations r>0.9, p<0.0001) [33]. Furthermore, we recently showed that self-reported waist circumference at follow-up could be used as a proxy for measured waist in regression analyses [48].We also used a prediction equation to improve the accuracy of self-reported anthropometry and this resulted in values for weight and waist circumference gain comparable to levels observed in the two EPIC centres with measured weight both at baseline and follow-up. It seems unlikely that the significant association with the health behaviour score is explained by inaccuracies in anthropometry changes. Rather, our results may be attenuated due to random measurement error associated with self-reported lifestyle factors. We also cannot rule out residual confounding by poorly and/or unmeasured confounders.

Another limitation is that lifestyle assessment for creating the score was only done at baseline and possible behavioural changes during follow-up were not taken into account. In participants for whom we had information on smoking status during follow-up, quitting smoking was associated with weight gain compared to stable smokers [14]. Consequently, excluding participants who smoked at baseline and quit smoking during follow-up resulted in an attenuation of the beneficial effects on weight and waist circumference gain for combined positive health behaviours. Nevertheless, being physically active, adherent to the mMDS and never-smoking still had positive effects on weight gain in men and gain of waist circumference in men and women during a 5-year follow-up. Since quitting smoking seems to lead mainly to a short-term weight gain [13], [14], [29], the effect of excluding quitters might diminish with a longer follow-up time. Future studies are warranted investigating the combined effect of lifestyle factors on weight and waist circumference gain particularly considering (long-term) changes of all health behaviours.

We constructed a pragmatic simple health behaviour score by using dichotomous variables where 1 point was given for the presence of each of the positive health behaviours without weighting the individual strength of the relationship with anthropometry. A weighted approach might improve the estimation of the effects of the individual score, but the combined effect of reporting all three healthy behaviours would be similar. Nechuta et al. [23] showed that results using a more differentiated score were comparable to using the pragmatic score for the estimation of the association between combined lifestyles and mortality.

We developed a score including health behaviours that are presumable easy to achieve in the general population. We, for example, dichotomized physical activity into inactive (sedentary job and no recreational activity) and active (any category with activity levels above the latter). Therefore, a relatively high proportion of participants scored one or more points in our health behaviour score.

The choice of the components of our score was based on prior findings from EPIC-PANACEA. Therefore, we used the mMDS, which was associated with weight change [6], [12], as the healthy diet component. One may argue that the Mediterranean Diet is a reflection of the traditional dietary pattern in Mediterranean countries rather than an a-priori healthy diet index based on scientific evidence. However, it has extensively been shown to be linked to a decreased risk of several chronic diseases and mortality [49]. Also others used the Mediterranean Diet in their health behaviour score and showed that the score was related to lower mortality risk [16], [20]. It may well be possible that other healthy diets commonly used in Europe may also lead to similar favourable effects on weight and waist circumference gain.

Our results suggest that public health programs aiming at reducing the burden of obesity could benefit from targeting a cluster of behaviours. We show that combining health behaviours was associated to a lower gain in waist circumference – a valid marker of central adiposity [50] - independent of changes in BMI. Prevention of waist circumference gain is of potential importance because abdominal obesity, independent of general obesity, appears to be directly related to total mortality [2].

In conclusion, the combination of three positive health behaviours was associated with significantly lower 5-year weight and waist circumference gain compared with participants who scored to be inactive, smoking and low adherent to the Mediterranean Diet.

## Supporting Information

Figure S1 Country/Centre-specific association between the health behaviour score (highest category, i.e. all three health behaviours) and 5-year weight change (kg) in men. Country or centre specific estimates were calculated using general linear models in centres and countries with one centre only, or multilevel mixed-effects linear regression models in countries with more than one centre, and were adjusted age, total energy intake, baseline body mass index, education, alcohol intake and follow-up time. The overall estimate was calculated using random effect meta-analyses. Because of differences in assessment of follow-up weight, and/or different follow-up times, the centres in Germany (Heidelberg, Potsdam), United Kingdom (Cambridge, Oxford-General population, Oxford-Health Conscious) and the Netherlands (Utrecht, Doetinchem, Amsterdam/Maastricht) were treated as separate cohorts.
(TIF)

Figure S2 Country/Centre-specific association between the health behaviour score (highest category, i.e. all three health behaviours) and 5-year weight change (kg) in women. Country or centre specific estimates were calculated using general linear models in centres and countries with one centre only, or multilevel mixed-effects linear regression models in countries with more than one centre, and were adjusted age, total energy intake, baseline body mass index, education, alcohol intake and follow-up time. The overall estimate was calculated using random effect meta-analyses. Because of differences in assessment of follow-up weight, and/or different follow-up times, the centres in Germany (Heidelberg, Potsdam), United Kingdom (Cambridge, Oxford-General population, Oxford-Health Conscious) and the Netherlands (Utrecht, Doetinchem, Amsterdam/Maastricht) were treated as separate cohorts.
(TIF)

Figure S3 Country/Centre-specific association between the health behaviour score (highest category, i.e. all three health behaviours) and 5-year waist circumference change (cm) in men. Country or centre specific estimates were calculated using general linear models in centres and countries with one centre only, or multilevel mixed-effects linear regression models in countries with more than one centre, and were adjusted age, total energy intake, baseline body mass index, baseline waist circumference, education, alcohol intake and follow-up time. The overall estimate was calculated using random effect meta-analyses.
(TIF)

Figure S4 Country/Centre-specific association between the health behaviour score (highest category, i.e. all three health behaviours) and 5-year waist circumference change (cm) in women. Country or centre specific estimates were calculated using general linear models in centres and countries with one centre only, or multilevel mixed-effects linear regression models in countries with more than one centre, and were adjusted age, total energy intake, baseline body mass index, baseline waist circumference, education, alcohol intake and follow-up time. The overall estimate was calculated using random effect meta-analyses.
(TIF)
