# Supplementary figures and images for: Combined Impact of Lifestyle Factors on Prospective Change in Body Weight and Waist Circumference in Participants of the EPIC-PANACEA Study
Source: PLoS One. 2012 Nov 30;7(11):e50712. doi: 10.1371/journal.pone.0050712 (PMC3511344; doi:10.1371/journal.pone.0050712)

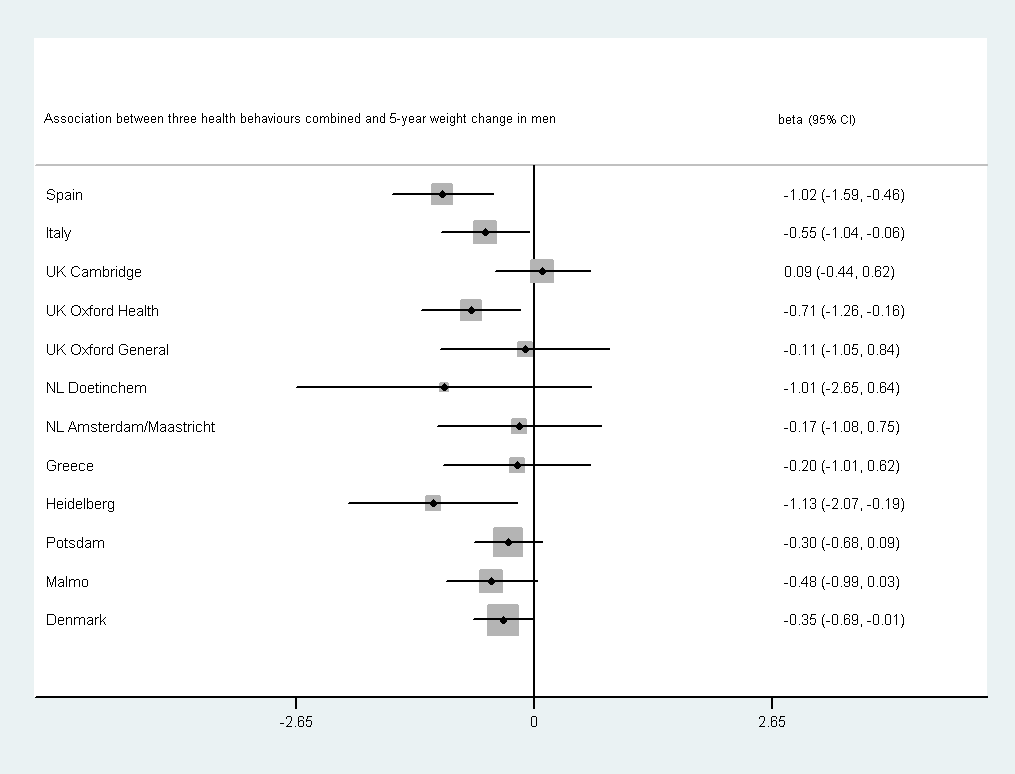

Supplement: Figure S1 — Country/Centre-specific association between the health behaviour score (highest category, i.e. all three health behaviours) and 5-year weight change (kg) in men. Country or centre specific estimates were calculated using general linear models in centres and countries with one centre only, or multilevel mixed-effects linear regression models in countries with more than one centre, and were adjusted age, total energy intake, baseline body mass index, education, alcohol intake and follow-up time. The overall estimate was calculated using random effect meta-analyses. Because of differences in assessment of follow-up weight, and/or different follow-up times, the centres in Germany (Heidelberg, Potsdam), United Kingdom (Cambridge, Oxford-General population, Oxford-Health Conscious) and the Netherlands (Utrecht, Doetinchem, Amsterdam/Maastricht) were treated as separate cohorts. (TIF) [file pone.0050712.s001.tif]

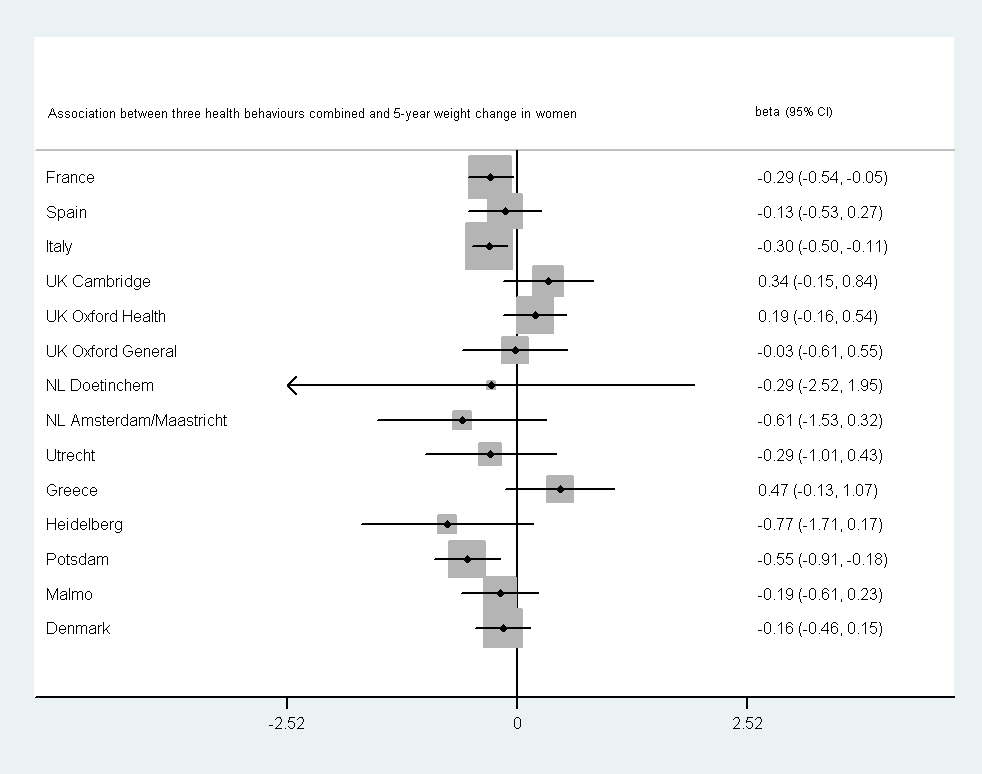

Supplement: Figure S2 — Country/Centre-specific association between the health behaviour score (highest category, i.e. all three health behaviours) and 5-year weight change (kg) in women. Country or centre specific estimates were calculated using general linear models in centres and countries with one centre only, or multilevel mixed-effects linear regression models in countries with more than one centre, and were adjusted age, total energy intake, baseline body mass index, education, alcohol intake and follow-up time. The overall estimate was calculated using random effect meta-analyses. Because of differences in assessment of follow-up weight, and/or different follow-up times, the centres in Germany (Heidelberg, Potsdam), United Kingdom (Cambridge, Oxford-General population, Oxford-Health Conscious) and the Netherlands (Utrecht, Doetinchem, Amsterdam/Maastricht) were treated as separate cohorts. (TIF) [file pone.0050712.s002.tif]

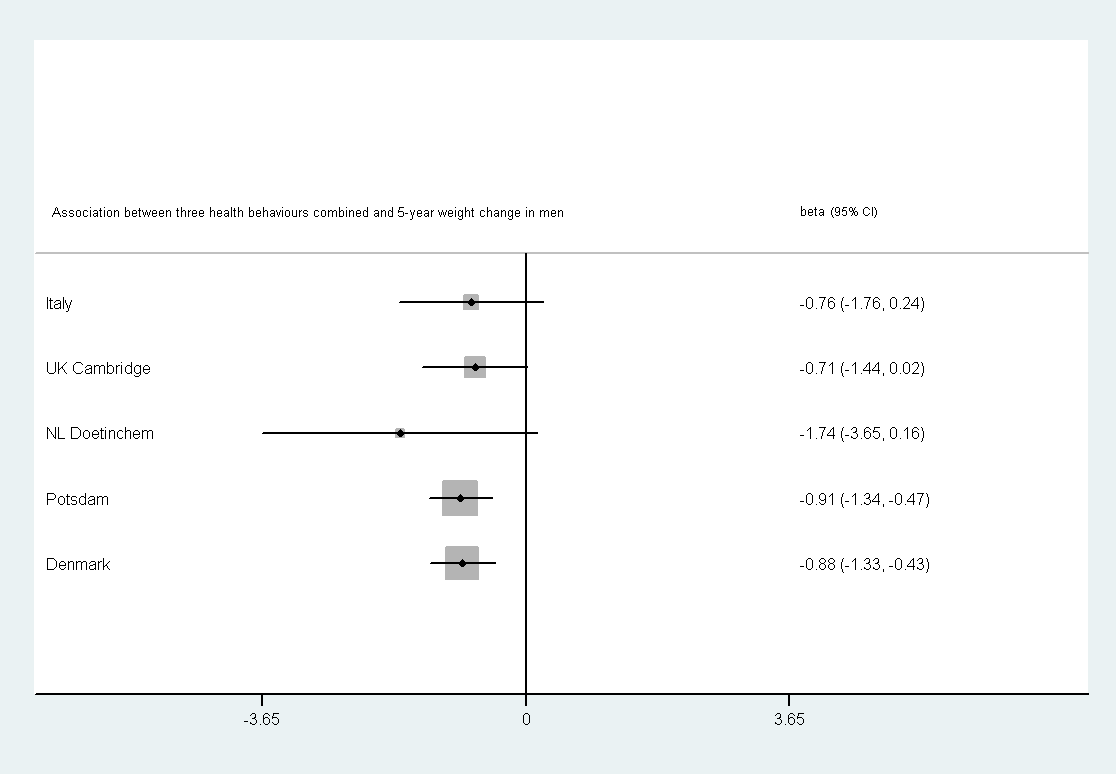

Supplement: Figure S3 — Country/Centre-specific association between the health behaviour score (highest category, i.e. all three health behaviours) and 5-year waist circumference change (cm) in men. Country or centre specific estimates were calculated using general linear models in centres and countries with one centre only, or multilevel mixed-effects linear regression models in countries with more than one centre, and were adjusted age, total energy intake, baseline body mass index, baseline waist circumference, education, alcohol intake and follow-up time. The overall estimate was calculated using random effect meta-analyses. (TIF) [file pone.0050712.s003.tif]

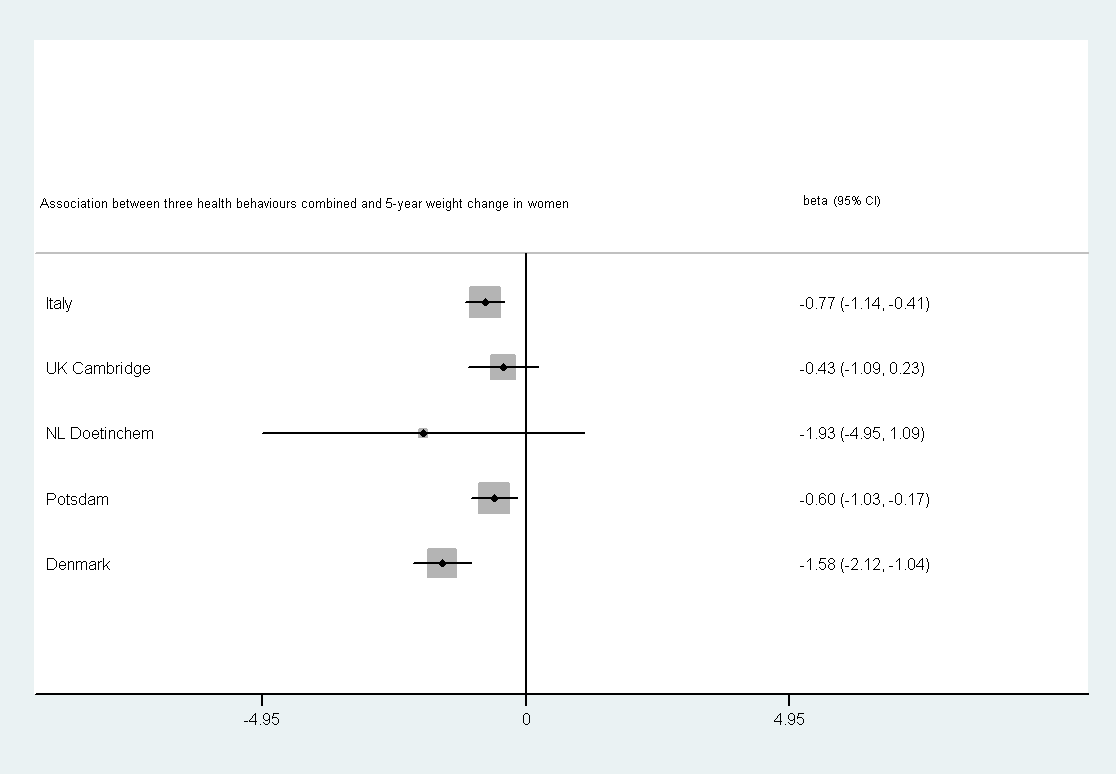

Supplement: Figure S4 — Country/Centre-specific association between the health behaviour score (highest category, i.e. all three health behaviours) and 5-year waist circumference change (cm) in women. Country or centre specific estimates were calculated using general linear models in centres and countries with one centre only, or multilevel mixed-effects linear regression models in countries with more than one centre, and were adjusted age, total energy intake, baseline body mass index, baseline waist circumference, education, alcohol intake and follow-up time. The overall estimate was calculated using random effect meta-analyses. (TIF) [file pone.0050712.s004.tif]
